# Polydatin, a natural precursor of resveratrol, induces cell cycle arrest and differentiation of human colorectal Caco-2 cell

**DOI:** 10.1186/1479-5876-11-264

**Published:** 2013-10-20

**Authors:** Salvatore De Maria, Ilaria Scognamiglio, Angela Lombardi, Nicola Amodio, Michele Caraglia, Maria Cartenì, Gianpietro Ravagnan, Paola Stiuso

**Affiliations:** 1 Operative Unit of Naples GLURES, Academic SPIN-OFF of Ca’ Foscari University of Venice, Venice, Italy; 2 Institute “Massimo D’Azeglio”, Marano di Napoli, Naples 80016, Italy; 3 Department of Biochemistry, Biophisics and General Pathology, Second University of Naples, Naples, Italy; 4 Department of Experimental and Clinical Medicine, Magna Graecia University of Catanzaro, Catanzaro, Italy; 5 Department of Molecular Sciences and Nanosystems, Ca’ Foscari Venezia University, Venice, Italy

**Keywords:** Human colon carcinoma, hsp27, Differentiation

## Abstract

**Background:**

Human colon adenocarcinoma cells are resistant to chemotherapeutic agents, such as anthracyclines, that induce death by increasing the reactive oxygen species. A number of studies have been focused on chemo-preventive use of resveratrol as antioxidant against cardiovascular diseases, aging and cancer. While resveratrol cytotoxic action was due to its pro-oxidant properties. In this study, we investigate whether the Resveratrol (trans-3,5,49-trihydroxystilbene) and its natural precursor Polydatin (resveratrol-3-O-b-mono- D-glucoside, the glycoside form of resveratrol) combination, might have a cooperative antitumor effect on either growing or differentiated human adenocarcinoma colon cancer cells.

**Methods:**

The polydatin and resveratrol pharmacological interaction was evaluated *in vitro* on growing and differentiated Caco-2 cell lines by median drug effect analysis calculating a combination index with CalcuSyn software. We have selected a synergistic combination and we have evaluated its effect on the biological and molecular mechanisms of cell death.

**Results:**

Simultaneous exposure to polydatin and resveratrol produced synergistic antiproliferative effects compared with single compound treatment. We demonstrated that polydatin alone or in combination with resveratrol at 3:1 molar ratio synergistically modulated oxidative stress, cell cycle, differentiation and apoptosis. Worthy of note treatment with polydatin induced a nuclear localization and decreased expression of heat shock protein 27, and vimentin redistributed within the cell.

**Conclusions:**

From morphological, and biochemical outcome we obtained evidences that polydatin induced a transition from a proliferative morphology to cell-specific differentiated structures and caused human CaCo-2 cell death by induction of apoptosis. Our data suggest the potential use of polydatin in combination chemotherapy for human colon cancer.

## Introduction

Colorectal cancer (CRC) remains a leading cause of mortality among many racial and ethnic groups throughout the world [1-3]. Adenocarcinoma cells, such as CRC cells, are remarkably resistant to damage induced by radiation or systemic, immunological and chemotherapeutic agents. As a consequence, the tumours are hard to treat and often proliferate rapidly, even under conditions that may adversely affect normal cells. The mechanisms underlying its survival advantage may be related in part to the high endogenous expression of stress proteins. In contrast to normal cells, the basal level of inducible heat shock proteins (HSPs) are frequently higher in tumour cells [4,5] Phytochemicals are among the most promising chemopreventive and treatment options for the management of cancer. The ideal characteristics or chemopreventive/therapeutic agents is the specific modulation of aberrant signalling pathways through the induction of apoptosis [6]. Resveratrol (trans-3,5,49-trihydroxystilbene) and its natural precursor Polydatin (resveratrol-3-O-b-mono- D-glucoside Product Origin: Root of Polygonum cuspidatum) are phytoalexins, molecules produced by spermatophytes plants to protect germinal centers, fruits, and roots, by attack by fungi, bacteria or free radicals [7]. Chemically these molecules are stilbenes derivatives. In the Resveratrol there are 3 hydroxyl-groups in 3, 4, and 5 position of stilbene scaffold whereas Polydatin has the position 3 is occupied by a glucopiranoside ring. Substitution of position 3 with a sugar molecule do as not interfere with scavenger functions of the hydroxi-stilbene (ISBs) that is ascribed in major measure to 4′ OH. Wallerath *et al.*[8] reported that resveratrol might rapidly increase NO production in cultured endothelial cells. At physiologic concentrations, NO protects the gastrointestinal mucosa from injury. By acting as an antioxidant, inhibiting leukocyte adherence, and maintaining mucosal blood flow [9,10]. During inflammation, intestinal epithelial cells are exposed to cytokines, bacterial products, and many other substances that affect cellular functions [11]. Under these conditions, NO synthase II is induced in a variety of cells including the intestinal epithelium. NO synthase II, is capable of generating high local intracellular and extracellular concentrations of NO [12-14]. As part of the inflammatory process or sepsis, activated inflammatory cells generated large amounts of superoxide anions (O_2_^-^). Abundant NO and O_2_^-^ radicals react rapidly to form peroxynitrite (ONOO^-^), an extremely reactive and toxic molecule [15-17]. Peroxynitrite is capable of nitrating the tyrosine residues of proteins, thereby disrupting cellular signalling systems that depend upon tyrosine phosphorylation [18,19]. Dietary polyphenols with phenol rings are metabolized by peroxidase to form pro-oxidant phenoxy radicals which are sufficiently reactive to co-oxidize GSH or NADH accompanied by extensive oxygen uptake and ROS formation. The aim of this work is to investigate the *in vitro* antioxidation and antiproliferative effects of polydatin and resveratrol alone or in combination in human colon adenocarcinoma CaCo-2 cells. This cell line is not particularly sensitive to treatment with chemotherapeutic agents, that induce death by oxidative stress. Moreover, as differentiated Caco2 cells is a well-accepted model for human enterocytes, they have been used to characterize a safety profile of compounds in terms of cell selectivity [20,21]. Moreover, we investigated the effects of polydatin and resveratrol and its combination on colon adenocarcinoma cell lines in terms of growth and apoptosis, cell cycle differentiation and modulation of HSP27, iNOS and vimentin intracellular level and distribution.

## Material and methods

### Chemicals

All chemicals, of the highest available quality, were obtained from Sigma Chemical Co. (St. Louis, USA). Trans-polydatin and trans-resveratrol, with a purity grade higher than 99%, have been supplied by Ghimas spa (Casalecchio, Bologna, Italy). The compounds were prepared in according to the method described in patent EP 1 292 319 B1 and EP 1 292 320 [22,23].

### Cell culture

Caco-2 (American Type Culture Collection, Rockville, MD, USA), was grown at 37°C in h-glucose MEM containing: 1% (by vol) non-essential amino acids and supplemented with 10% (by vol) de-complemented fetal bovine serum (FBS) (Flow, McLean, VA, USA), 100 U · mL-1 penicillin, 100 mg · mL-1 streptomycin, 1% L-glutamine and 1% sodium pyruvate. Cells were grown (17–21 passages) in a humidified atmosphere of 95% air/5% CO2 at 37°C, and in six multi-well plates at different cell densities. After incubation for 4 h in Dulbecco’s modified Eagle’s medium (DMEM) with 10% FBS, the cells were washed with 1% phosphate-buffered saline (PBS) to remove unattached dead cells, and were incubated with different concentrations of trans-resveratrol (trans-3,5,4′-trihydroxystilbene) and trans-polydatin (trans-5,4′-dihydroxystilbene-3-O-β-D-glucopyranoside). All experiments were performed in triplicate.

### Sensitivity of the cell lines to hydroxystilbenes (ISBn)

We assessed the sensitivity of the cell lines tested to ISBn using a microplate colorimetric assay that measures the ability of viable cells to transform a soluble tetrazolium salt (MTT) to an insoluble purple formazan precipitate. Cells were plated at the appropriate density (5 × 10^3^ undifferentiated Caco-2 cells per well and 20 × 10^3^ differentiated Caco-2 cells per well) in 96-well microtitre plates. After 4 h, cells were exposed to various concentrations of ISBn for 24 h. Then, 50 μL of MTT (1 mg · mL-1) and 200 μL of medium were added to the cells in each well. After a 4 h incubation at 37°C, the medium was removed, then the formazan crystals were solubilized by adding 150 μL of DMSO and by mixing it in an orbital shaker for 5 min. Absorbance at 550 nm was measured using a plate reader. Experiments were performed in triplicate. As a control, 0.5% DMSO was added to untreated cells.

### ISBn combination studies

For the study of the synergism between trans-polydatin and trans – resveratrol on growth inhibition of Caco-2 cells, the cells were seeded in 96-multiwell plates at the appropriate density (5 × 10^3^ undifferentiated Caco-2 cells per well and 20 × 10^3^ differentiated Caco-2 cells per well). After 24 h incubation at 37°C the cells were treated with different concentrations of polydatin (from 0 to 500 μM) or resveratrol (from 0 to 500 μM) and their combinations (50:50, 75:25, 25:75 molar ratio pol:res respectively). Drug combination studies were based on concentration-effect curves generated as a plot of the fraction of unaffected (surviving) cells vs. drug concentration after 24 h of treatment. Assessment of synergy was performed quantitating drug interaction by the Calcusyn computer program (Biosoft, Ferguson, MO). Combination index (CI) values of <1, 1, and >1 indicate synergy, additivity, and antagonism, respectively [15]. Furthermore, we analyzed the specific contribution of trans-polydatin and trans – resveratrol on the cytotoxic effect of the combination by calculating the potentiation factor (PF), defined as the ratio of the IC50 of either trans-polydatin or trans – resveratrol alone to the IC50 of polydatin/resveratrol combinations, respectively, as described before; a higher PF indicates a greater cytotoxicity.

### Alkaline phosphatase (ALP) activity

ALP activity was used as marker of the degree of differentiation of Human CaCo-2 cells. Attached and floating cells were washed and lysed with 0.25% sodium deoxycholate, essentially as described by Herz *et al.*[24]. ALP activity was determined using Sigma Diagnostics ALP reagent (no. 245). Total cellular protein content of the samples was determined in a microassay procedure as described by Bradford [25] using the Coomassie protein assay reagent kit (Pierce). ALP activity was calculated as units of activity per milligram of protein.

### Nitrite assays

NO is rapidly converted into the stable end products nitrite and nitrate. Nitrite was measured by the Griess reaction as reported by Green *et al.*[26]. The nitrite assay used in this work were described in Gomez-Monterrey I. *et al.*

### Flow cytometry analysis

Caco-2 cells were seeded in six multi-well plates at the density of 25 × 10^5^ cells per plate. After 24 h of incubation with ISBn cells were washed in PBS, centrifuged and directly stained in a propidium iodide (PI) solution (50 mg PI in 0.1% sodium citrate, 0.1% NP40, pH 7.4) for 30 min at 4°C in the dark. Flow cytometric analysis was performed using a FACScan flow cytometer (Becton Dickinson, San Jose, CA, USA). To evaluate cell cycle, PI fluorescence was collected as FL2 (linear scale) by the ModFIT software (Becton Dickinson). For the evaluation of intracellular DNA content, not less than 20 000 events for each point were analysed in at least three separate experiments giving a SD less than 5%. Superoxide anion production in mitochondria was determined by hydroethidine (HE) staining. The treated and untreated cells were incubated for 1 h with 20 ng · mL-1 HE, and were scraped, washed twice with PBS and the cell pellet was added to 1 mL PBS. HE–superoxide anion (HE-O) accumulation was measured by FACScan flow cytometer (FACScan, Becton Dickinson) using CellQuest software. For each sample 2 × 10^4^ events were acquired. Analysis was carried out in triplicate in at least three separate experiments.

### Immunostaining and confocal microscopy

Caco-2 cells grown were fixed in PBS 4% paraformaldehyde then permeabilized 5 min with PBS 1% Triton. Immunostaining was carried out by incubation with anti-iNOS, antiHsp27 and anti-vimentin antibodies 1:1000 followed by revelation using Cy3-conjugated anti-rabbit immunoglobulin (Ig) G antibodies (Jackson Immunoresearch Laboratories, West Grove, PA) at a dilution of 1/200 for 45 minutes. The cells were analyzed by an LSM-410 Zeiss confocal microscope.

### Evaluation of apoptosis by Western blot analysis

CaCo-2 cells were grown for 24 h with or without Resveratrol and/or polidatin in the previously described experimental conditions. For cell extract preparation, cells were washed twice with ice-cold PBS/BSA, scraped and centrifuged for 30 min at 4°C in 1 ml of lysis buffer (1% Triton, 0.5% sodium deoxycholate, 0.1 M NaCl, 1 mM EDTA, pH 7.5, 10 mM Na_2_HPO_4_, pH 7.4, 10 mM PMSF, 25 mM benzamidin, 1 mM leupeptin, 0.025 U/ml aprotinin). Equal amounts of cell proteins were separated by SDS-PAGE. The proteins on the gels were electro-transferred to nitrocellulose and reacted with the different MAbs.

### Transient transfections

Cells were seeded at a density of 3 × 10^5^ cells per well in 6-well dishes (∼70% confluency) and transfected in triplicate using the Transfection Reagent according to the manufacturer’s protocol as previously described [27].

The pcDNA.3.1- HA-myr-AKT-dominant active construct or the empty pcDNA-em-GFP vectors used in this work were described in Amodio N *et al.*[27]. Experiments were repeated at least three times.

### Statistical analysis

Values are expressed as the mean ± SE. The significance of the difference between the control and each experimental test condition was analysed by unpaired Student’s *t*-test, and P < 0.05 was considered statistically significant.

## Results

### Effect of hydroxy-trans-stilbene (ISBn) in Caco-2 cell lines

**Figure 1:**
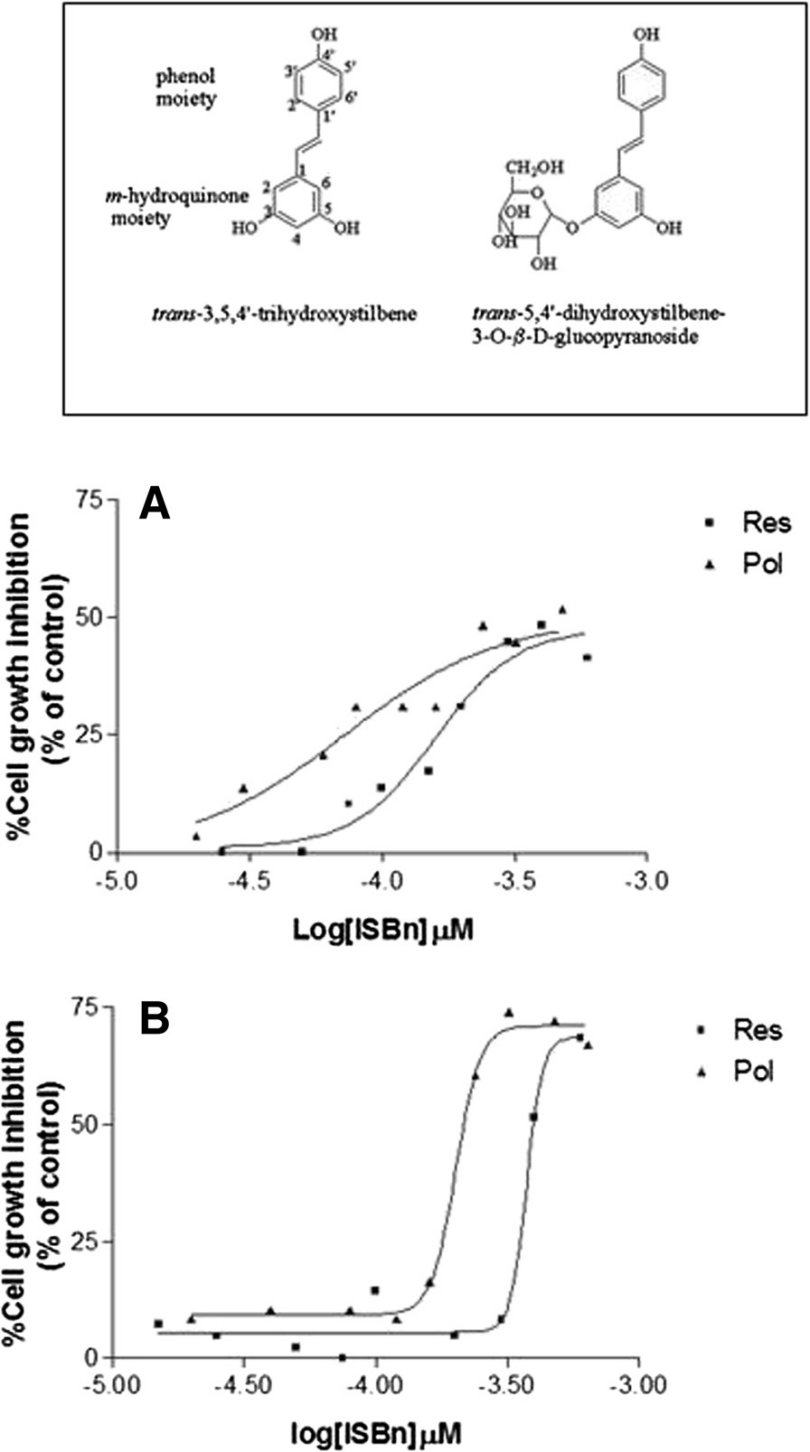
**Effects of polydatin and resveratrol on cell growth of growing and differentiated Caco-2 cells.** Growing Caco-2 cells **(**panel **A)** and differentiated Caco-2 cells **(**panel **B)** were treated for 24 h with increasing concentrations (0.2-300 μM) of polydatin and resveratrol. The effects on the cell growth were expressed as % inhibition respect to the untreated cells (control). Cell growth was evaluated by the MTT assay as described in Materials and Methods. In the inset was reported the structure of trans-resveratrol and its natural precursor polydatin.

We evaluated the effects of both trans-polydatin (Pol) and trans-resveratrol (Res) on the growth inhibition of undifferentiated (exponentially growing) and differentiated (post-confluent) Caco-2 cell lines (Figure 1). Both ISBn induced a dose-dependent growth inhibition at 24 h with 72 and 192 μM (IC50) of Pol in growing and differentiated cells respectively, while the IC50 in growing and differentiated Caco-2 cells were 156 and 373 μM, of Res concentrations respectively (Table 1). The antiproliferative effect result higher in the undifferentiated CaCo-2 cells compared to the differentiated cells for both ISBn, moreover the polydatin resulted more cytotoxic respect to resveratrol in both cellular model.

**Table 1:** Effect of polydatin (Pol), resveratrol (Res), on growth of pre- and post-confluent Caco-2 cells

**Compound**	**IC50 (μM) Pre-conf**	**IC50 (μM) Post-conf**
Res	156	373
Pol	72	192

### Synergistic antiproliferative effect of polydatin/resveratrol combination

**Figure 2:**
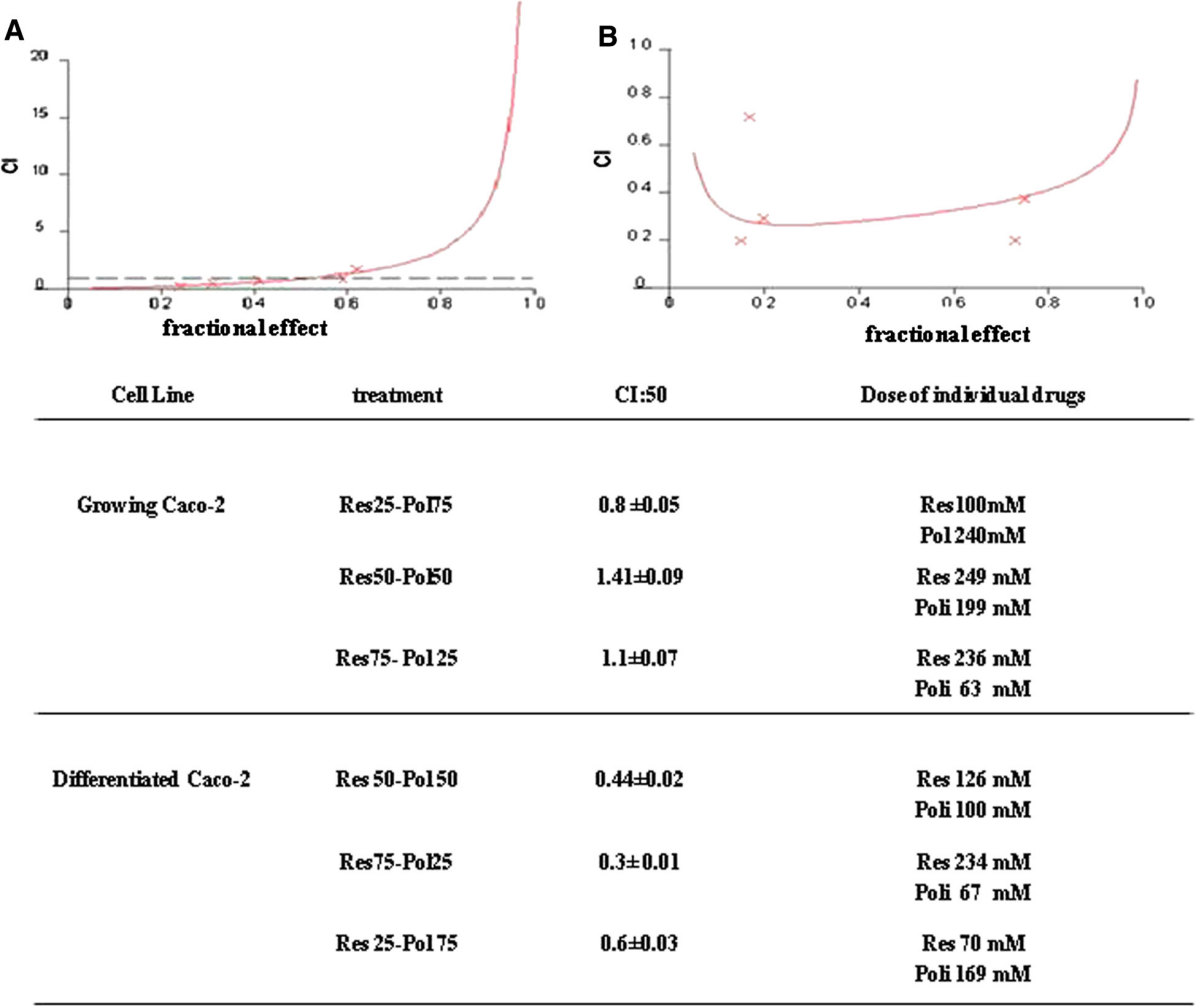
***In vitro *synergistic antiproliferative effect of ISBn combination on growing and differentiated Caco-2 cells.** CI/fractional effect curves showed the CI versus the fraction of cells affected/killed by polydatin and resveratrol in combination for the growing **(**panel **A)** and differentiated Caco-2 cell lines **(**panel **B)**. Combination analysis was done using the method described by Chou and Talalay (see Materials and methods). Combinations were synergistic when CI were <0.8. Representative experiment carried out at least twice for each cell line; in the figure the mean CI50 were reported.

On the basis of these results, we have evaluated if the ISBn could be synergistic in inducing cell growth inhibition of Caco-2 cells. Specifically, we evaluated the growth inhibition induced by different concentrations of pol/res combination at 24 h on Caco-2 cell line. We used Calcusyn [28], a dedicated software, to examine the synergism of our treatments. With this software, synergistic conditions occur when the combination index (CI) is <1.0. When CI is <0.5 the combination is highly synergistic. We found that the combination of Pol and Res was synergistic when the two compounds were used at higher concentrations of polydatin (pol/res molar ratio = 3:1) on growing Caco-2 cells (Figure 2A). On the other hand, antagonism was recorded when ratios with higher concentrations of Res were used (data not shown). In synergistic drug combination the CI50 (the combination index calculated for 50% cell survival by isobologram analysis) was 0.88. While all Pol/Res combinations at different molar ratios were highly synergistic on differentiated Caco-2 (Figure 2 panel B). These results demonstrate that the synergism on growing Caco-2 cells is only when the ratio polydatin/resveratrol is 3:1, whereas in the differentiated Caco-2 a very strong synergism can be recorded on cell proliferation when the concentration of resveratrol is higher. All subsequent evaluations were performed at 240 and 100 μM of Pol and Res respectively (3:1 polydatin/resveratrol molar ratio).

### ISBn combination induces cell cycle arrest and differentiation of growing Caco-2 cells

**Figure 3:**
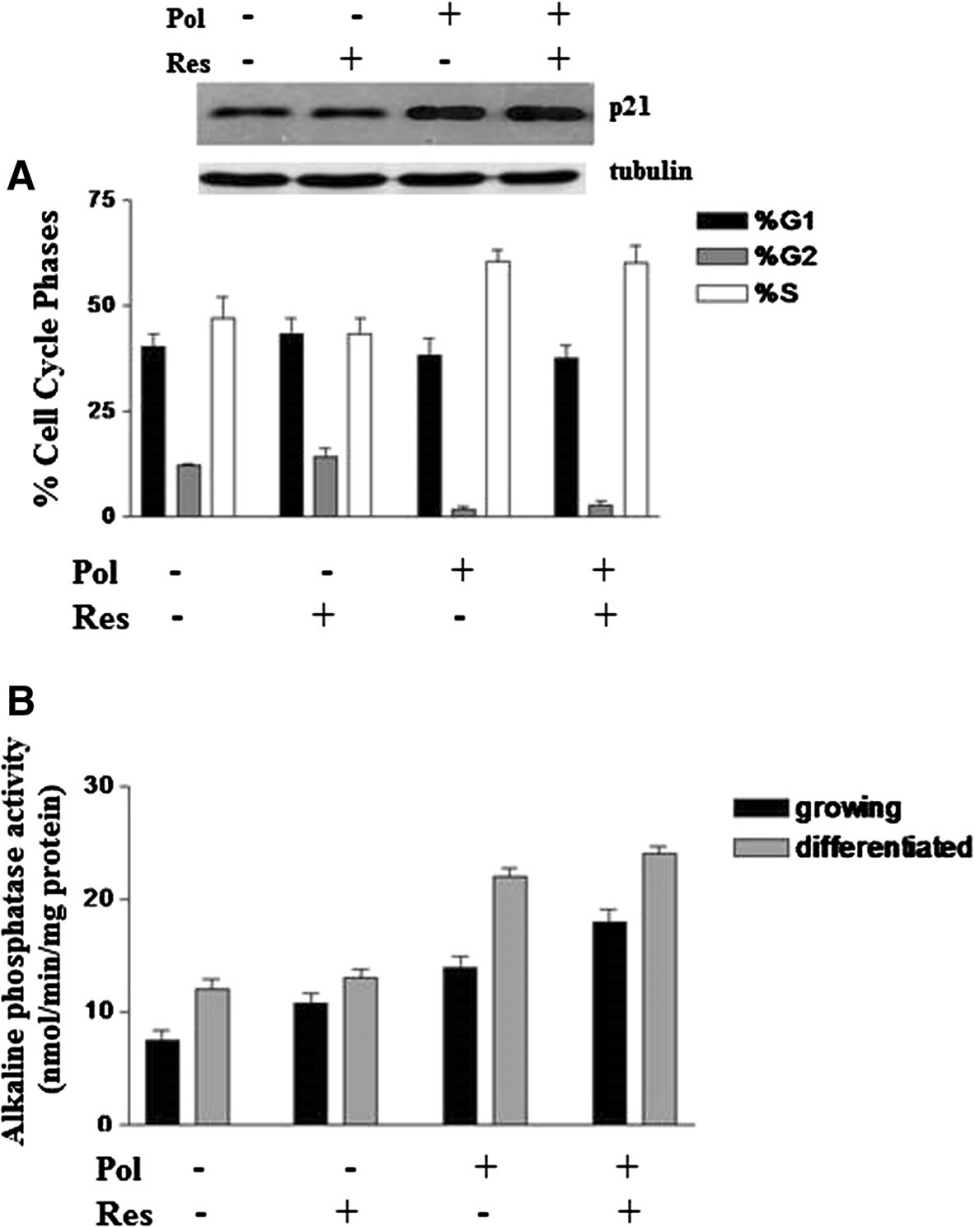
**A Effect of ISBn alone or in combinations on the distribution of growing Caco-2 cell populations.** Data represent the percentage of cells in each phase of the cell cycle. Cell cycle distribution was determined by DNA flow cytometric analysis. Samples from exponentially growing Caco-2 cells were analysed after 24 h of treatment with 100 and 240 μM of resveratrol and polydatin alone or in combinations. Numbers indicate percentage of cells in G0/G1, S and G2/M phases. Data are representative of four separate analyses. In the inset of panel A was reported the western blot analysis of p21 protein and tubulin expression in growing. **B**: Effect of ISBn on of Caco-2 cells differentiation. The differentiation of Caco-2 cells assessed by measurement of ALP activity after 24 h of culture with 0, 240, and 100 μM of Pol, Res and their combination. Summary data shown are means ± SEM; (*n* = 4; *P < 0.05).assessed by measurement of ALP activity.

The effect of ISBn on cell proliferation could be due to their actions on cell cycle and the induction of programmed cell death. In order to explore this possibility, we treated exponentially growing Caco-2 cells with polydatin and resveratrol alone or in combination and assessed its effects on cell cycle (Figure 3). The cells treated with polydatin alone or in combination with resveratrol for 24 h showed a 27% increase in the number of Caco-2 cells in S-phase, while G1 populations decreased. This finding was additionally confirmed by increase of p21 expression in Caco-2 cells treated with ISBn combination. As cell division arrest is one of the prerequisites for cell differentiation [28], we determined the effect of ISBn on Caco-2 differentiation by measuring alkaline phosphatase (ALP) activity as a marker of differentiation into enterocytes correlated to post-confluent phase [29]. In growing CaCo-2 cells both polydatin and resveratrol slightly augment the ALP enzyme activity (Figure 3 panel B). Synergistic effect on ALP activity was recorder in 3:1 pol/res molar ratio where a maximum of 30% of increase was reached, compared to untreated cells. On the other hand, in the differentiated cells 250%, 70% and 300% ALP activity increase with polydatin and resveratrol alone, and ISBn combination treated cells, respectively.

### Antioxidant effect of ISBn in CaCo-2 cells

**Figure 4:**
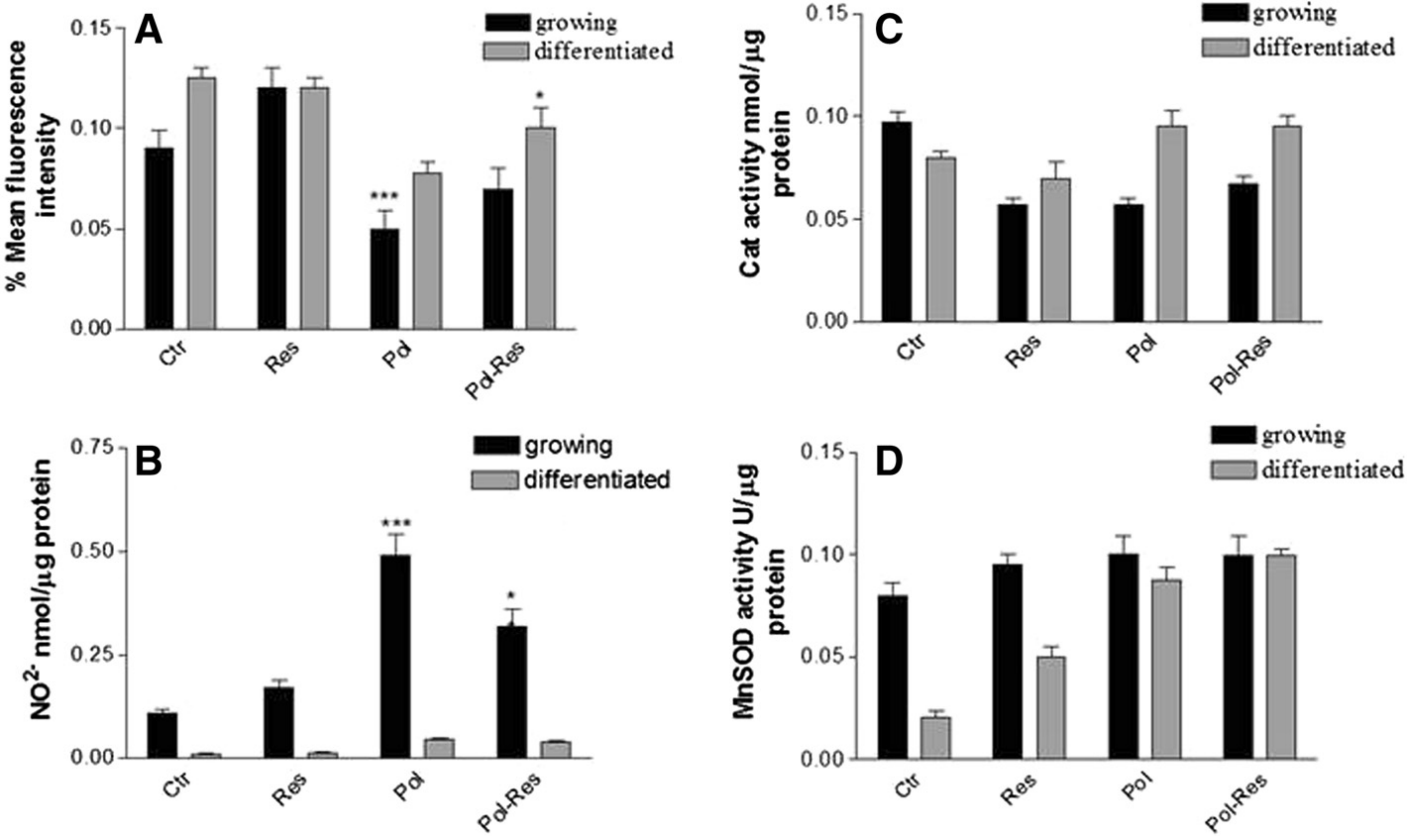
**Effect of ISBn alone or in combination on mithocondrial superoxide anions, nitric oxide, and enzyme scavengers activity in Caco-2 cells. (A)** After 24 h Caco-2 treatment with polydatin and resveratrol alone or in combination (240 and 100 μM) at 37°C, the mitochondrial superoxide anion production was analysed by HE (20 ng · mL-1) staining. Dye accumulation was analysed by FACScan flow cytometer (FACScan, Becton Dickinson) by the CellQuest software, and the intensities of the bands were expressed as percent control. For each sample, 2 × 10^4^ events were acquired. **(B)** Nitric oxide was measured in medium. Whole cell homogenates were used for evaluated the Catalase **(C)** and Mn-SOD activity **(D)**. The bars represent means ± SEM of three independent experiments. Asterisks indicate significant difference between the Caco-2-treated samples compared with control value **P < 0.003; *P < 0.05.

The antioxidant activity of resveratrol and polydatin alone or in combinations were studied evaluating the mithocondrial superoxide anions, extracellular NO production, and the scavenger enzymes. In Figure 4 were reported the mitochondrial superoxide anions (panel A), in growing and differentiated Caco-2 cells after 24 h of treatment with Pol, Res and their combination (molar ratio 3:1 Pol/Res). In growing CaCo-2 cells the polydatin determined of 2 fold a reductions of mitochondrial superoxide anions compared to untreated cells (ctr), whereas Res induced a weak increase of superoxide anions. In growing combination treatment Caco-2 cells the resveratrol mildly antagonized the reduction of superoxide anions induced by polydatin. The ISBs combinations induced a synergistic increase (p < 0.006) of TBARS (markers of lipid peroxidation) in the growing Caco-2 cells compared to untreated cells. In differentiated cells polydatin and resvertrol an about 4 and 2-fold increase of lipid peroxidatin, respectively, while the ISBn combination treatment induced an additional increase of TBARS values up to 5-fold compared to untreated cells (data not shown). The free NO production was determined in growth medium by measuring the stable oxidation products, NO_2_^-^. Only the polydatin caused an about 4 and 2-fold increased extracellular NO_2_^-^ production in growing and differentiated Caco-2 cells respectively (Figure 4B). On the other hand the ISBn combination treatment induced only a slight increase of NO_2_^-^ if compared to polydatin alone treatment, and this decrease was more evident in growing rather than in differentiated cells (Figure 4 panel B). The treatment of growing cells with ISBn alone or in combination induced a significantly decrease only of catalase activity (Figure 4 panel C). On the other hand, mainly polydatin induced a significantly increased of Mn-SOD activity in differentiated cells (Figure 4 panel D) compared to non treated control cells (CTR).

### Polydatin induced differentiation of pre-confluent cells by modulation and cellular localization of iNOS and HSP27 protein

**Figure 5:**
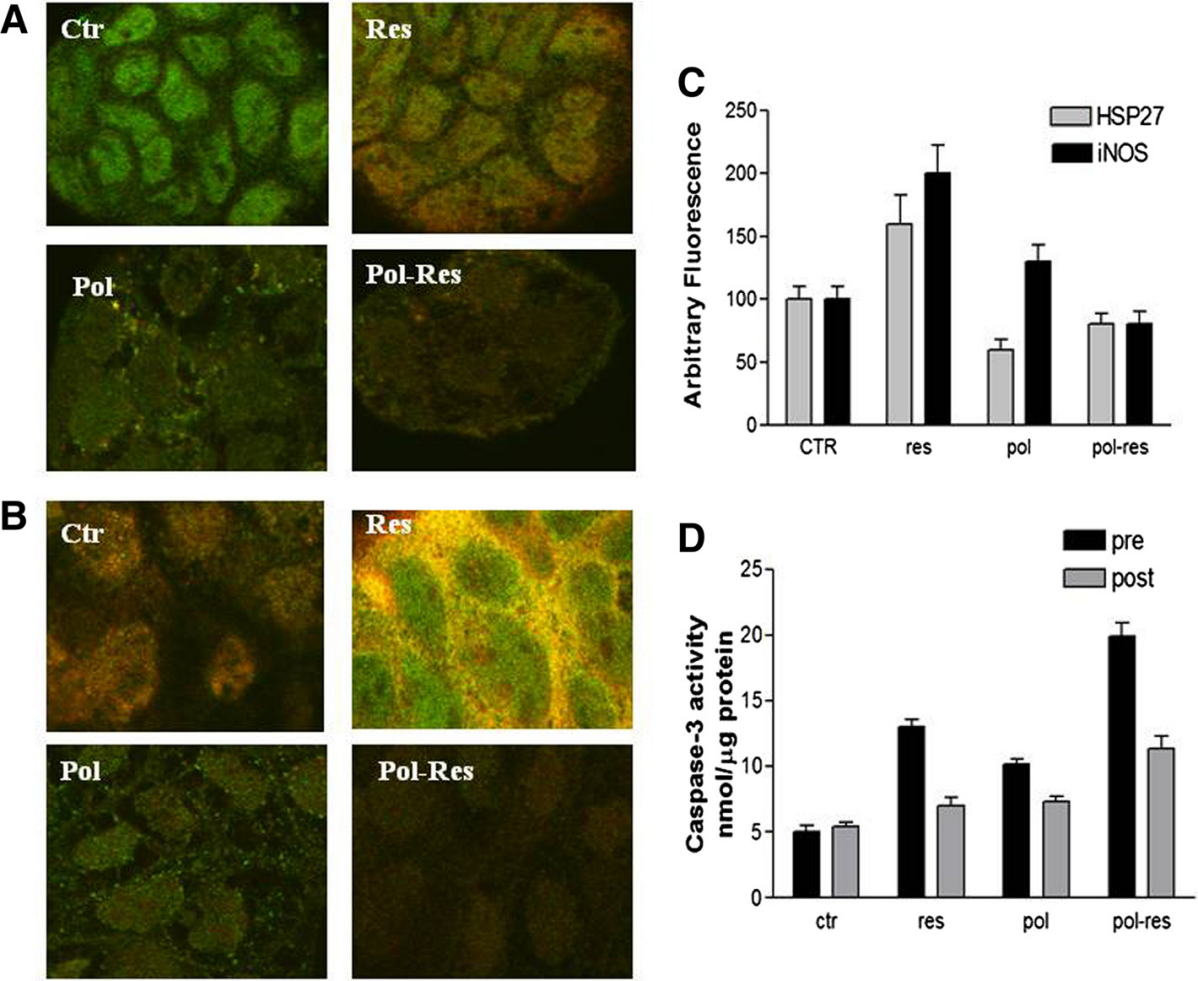
**Confocal analysis of ISBn treated growing Caco-2 cells stained for iNOS, hsp27, and vimentin.** Panel **A**: Subcellular co-localization of iNOS (red) and vimentin filaments (green) in Caco-2 cells without ISBn (ctr) and with Pol, Res, and Pol-Res combination treatment. The cells were incubated with anti-vimentin, anti-HSP27 and anti-iNOS antibodies. Images were obtained by confocal microscopy. Panel **B**: Subcellular co-localization of HSP-27 (red) and vimentin filaments (green) in Caco-2 cells without ISBn (ctr) and with Pol, Res, and Pol-Res combination treatment The cells were incubated with anti-vimentin, anti-HSP27 and anti-iNOS antibodies. Images were obtained by confocal microscopy. Panel **C**: The expression levels of the proteins are reported as intensities quantified with Image J software. Panel D: Caspase-3 activity on growing and differentiated Caco-2 cells without ISBn (ctr) and with Pol, Res, and Pol-Res combination treatment .Caspase-3 activity was determined by incubating whole-cell extracts with 40 μM caspase-3 substrate and measuring production of hydrolysed 7-amido-4-methyl-coumarin (AMC) groups using a multi-label plate reader. The results are representative of four separate experiments; summary data shown are means ± SEM.

iNOS induction is dramatically increased in postconfluent (differentiated) Caco-2 cells [30]. Level of iNOS expression in pre-confluent Caco2 cells was low if compared with ISBn-treated cells when analyzed by confocal microscopy (Figure 5 panel A). Polidatin induce iNOS cell localization in the citosol periphery whereas the enzyme was nuclear in resveratrol-treated cells. In growing Caco-2 cells treated with ISBn combination iNOS was accumulated both periphery and nuclear region. HSP27 immunostaining of preconfluent Caco2 cells showed an over-expression and cytoplasmic localization with and without resveratrol treatment (Figure 5 panel B). In contrast, polydatin treatment alone or in combination with resveratrol induced a reduces expression and nuclear localization of Hsp27 protein. At the same time, 24 h treatment with 240 microM polidatin induced a perinuclear redistribution and morphological rearrangement of Vimentin filaments.

### The synergistic effect of the ISBn combination on growth inhibition is mediated by apoptosis

**Figure 6:**
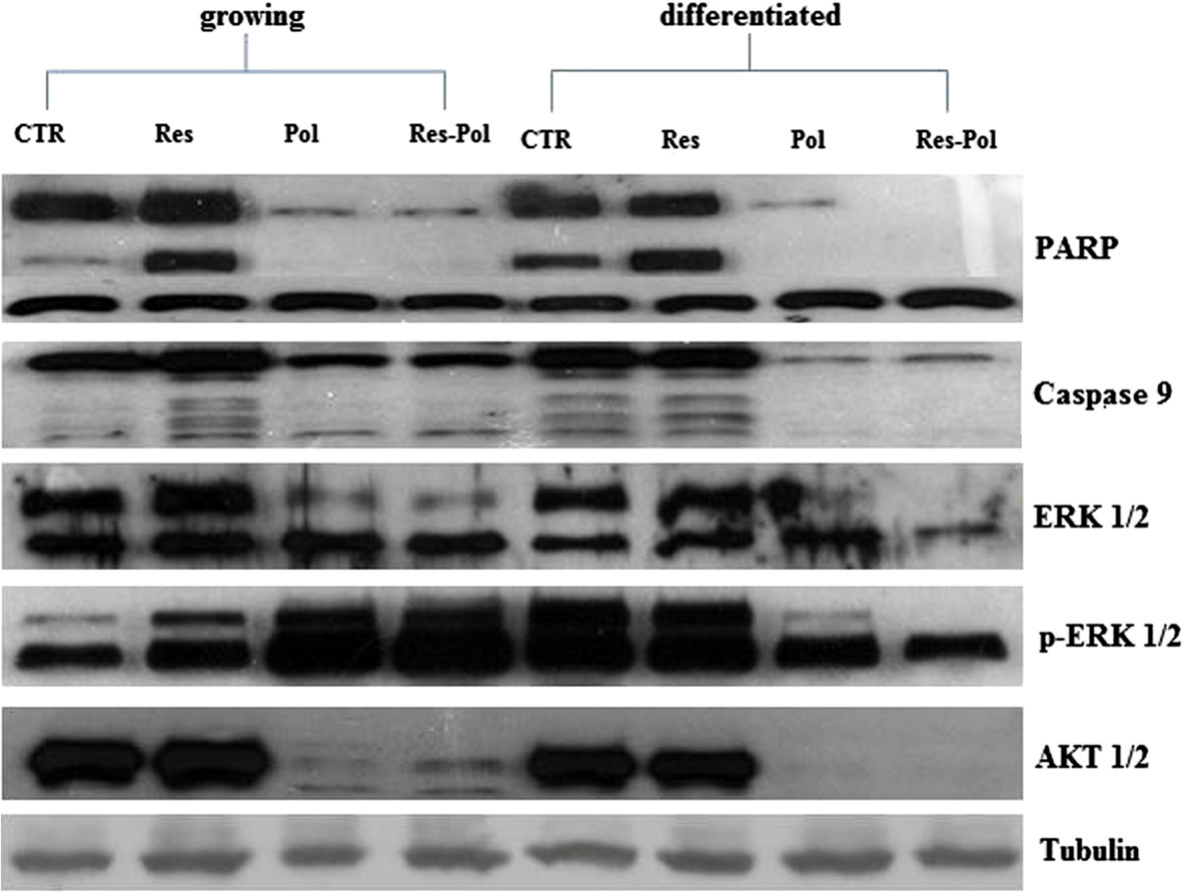
**Effects of ISBn treatment on PARP, Caspase 9, total ERK1/2, phosphorylated ERK1/2 (p-ERK), AKT, and tubulin expression evaluated by western blotting.** The pre and post-confluences Caco-2 cells were treated for 24h with ISBn alone or in combination. The bands associated with expression of PARP, Caspase 9, ERK1/2, phosphorylated ERK1/2 (p-ERK), AKT and house-keeping tubulin, after 24 h of treatment with ISBn are visualized. The expression of the house-keeping protein tubulin was used as loading control.

**Figure 7:**
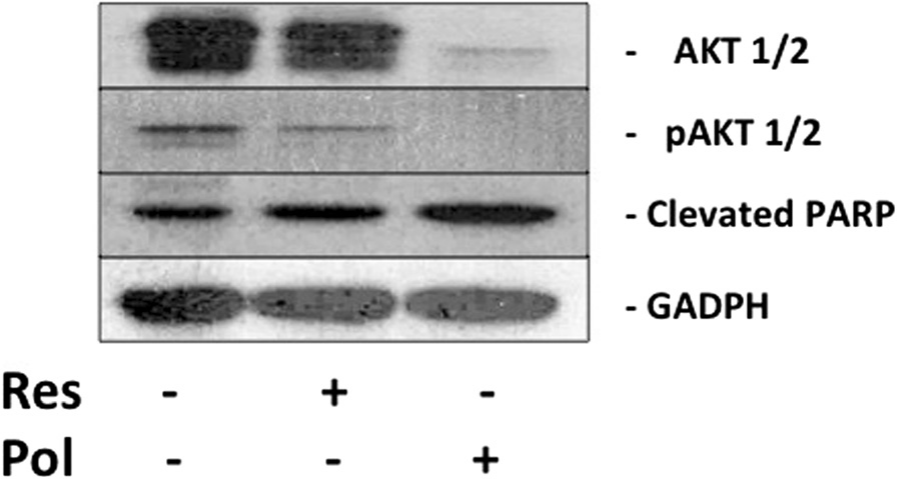
**Effect of ISBn treatment on expression of total AKT-1/2, Phospho-AKT-1/2 and cleaved PARP isoform in dominant positive Akt-transiently transfected CaCo2 cells (as described in “Materials and methods”).** The cells were treated with Pol and Res at their IC:50 concentrations for 24 h and then were processed for western blotting analysis as described in “Materials and methods”. GAPDH was used as lading control. Blots shown are from one representative experiment derived form three different experiments that gave always similar results.

Since HSP27 has been shown to inhibit cytochrome c-mediated activation of caspases [31], the apoptotic effect induced by ISB-treatment was investigated by analyzing caspase-3 activity. Caspase-3 activity was increased in growing Caco-2 cells treated with combination (Figure 5 panel D) [31-33]. In Caco-2 cells grown for 24 h with Resveratrol and Polydatin we found a marked cleavage of Poly(ADP-ribose) polymerase protein (PARP), paralleled a decrease of the full lengh isoform of the protein (Figure 6). The same data were recorded for caspase 9, a caspase initiator. We have evaluated the effects of resveratrol and polidatin on the terminal enzymes of the survival ras-dependent MAPK pathway, Erk-1 and Erk-2, and p-Erk1 and p-Erk2. We have found that Res alone induced a slight increase of Erk-1 and 2 paralleled a decrease their activities. Pol alone or in combination with Res induced a strong decrease of Erk-1 and Erk-2 expression and a strong increase of their activities both in preconfluent and post confluent cells, as evaluated with a western blotting assay using a mAb raised against the phosphorylated/activated isoforms of the two enzymes. (Figure 6). Thereafter, we have evaluated the effects of the single agents or the combination on another important survival pathway regulated by ras, the Akt/PKB signalling. In details, we have studied Akt expression with western blotting. We have found that Res alone did not induce significant changes in Akt expression while the Pol and the combination caused an almost total reduction of the expression of the enzyme (Figure 6). To demonstrate that polydatin could increase apoptosis by decreasing total AKT protein expression and function, we generated growing Caco-2 cells over-expressing AKT by transiently transfection with a full-length coding sequence of the dominant positive (constitutively activated) AKT gene vector. As shown in Figure 7, both total AKT and phosphorylated Akt were down-regulated in transiently transfected cells only after polydatin treatment. In te same experimental conditions, a concomitant increase of the cleaved form of PARP (Figure 7) was observed. All these data indicate that the synergistic effects of the polydatin and of Res/Pol combination in Caco-2 cells are paralleled by the disruption of two different survival pathways, and an increase of apoptosis*.*

## Discussion

The epithelium of the small intestine is a highly dynamic continuously renewed system, the whole process of proliferation, differentiation, apoptosis occurs over 3 to 5 days. Cells differentiation process, can be mimed *in vitro* using Caco-2 cell cultures. Although cancerous in origin, these cells undergo a gradual differentiation process that takes place spontaneously once confluence has been reached. In our previous studies we have investigated the ISBn immune-modulatory [34], anti-inflammatory [35], and anticancer [36-38] properties in melanoma [36], breast cancer [37] and colon cancer [38], derived cellular models. In the present study, we have shown that polydatin, alone or in combination with resveratrol induced cooperative antitumor effect on both growing and differentiated Caco-2 cell lines. In details, cell growth assays showed that the polydatin alone has a stronger cytotoxicity (IC50 = 72 μM) than Resveratrol (IC50 = 156 μM), in growing Caco-2 cells. The synergistic antiproliferative effect was reached only when the poly/res molar ratio was 3:1 as shown by the use of the median effect analysis and calculating CIs, while the combinations at 1 and 0.25 molar ratio were antagonistic. In differentiated Caco-2 cells the IC50 was reached at 192 and 373 μM of Pol and Res respectively, and the growth assay clearly showed that all pol/res molar ratio combinations were highly synergistic. The resistance of human colon adenocarcinoma cells to antineoplastic agents, that can induce cell death by oxidative stress are, at least in part related to the high endogenous expression of stress proteins, including the HSP family.

The literature contains extensive documentation for antioxidant (AO) action by resveratrol [39]; however the differential localization of resveratrol and polydatin in the lipid bilayer was also suggested [40] and can give an rationale to explain the synergistic effect observed. There is an apparent contradictions based on an appreciable number of reports providing evidence for pro-oxidant action of Res. Res, acting as AOs for lipids often have a pro-oxidant effect on DNA or protein. The effect of pro-oxidant Res activity, may lead to cell cycle arrest or apoptosis. In our experimental conditions, Res (100 μM) elicits pro-oxidant properties as evidenced by an increase of mithocondrial superoxide anion, not paralleled by cell cycle arrest and apoptosis. The Pol, on the other hand, at 240 μM induced after 24 h a significant mithocondrial superoxide anion reduction especially in growing Caco-2 cells, and this effect is attenuated by combination with Res. The reduction of ROS is not correlated with increased of, manganese superoxide dismutase (MnSOD) activity and was likely be due to direct antioxidant scavenger activity of polydatin. The decreased levels of mitochondrial superoxide anion was paralleled by increase of free NO production, suggesting a potential involvement of Pol in regulating the balance between NO and peroxynitrite. A protective effect of free NO has also been observed in hepatocarcinoma [41], HT-29 human colon carcinoma cell line [42], urinary bladder mucosa [43], and inflammatory cells [44]. Higher level of free NO may be responsible of dynamics of cytoskeleton [45] and cell differentiation [46]. Our finding suggests that the reductions in cell viability in growing Caco-2 cells elicited by polydatin was also associated to cell structural changes. We observed an increased differentiation evaluated by ALP activity with a concomitant redistribution of Vimentin and HSP27 proteins. The pre-confluent Caco-2 cell population surviving after Pol treatment (240 μM, 24 h), had a nuclear localization of hsp27, and an increase of cell number in S-phase. All these findings show that polydatin exerts cytotoxic activity through mechanisms of action different from resveratrol. Moreover, it is reasonable to suppose that the cause of polydatin-induced cell death was apoptosis, as suggested by activation of caspase-3 cystein protease, acting as a common effector pathway for apoptotic processes originating at both cell membrane and mitochondrial levels [47]. In fact we found that polydatin induced apoptosis in Caco-2 cells as a secondary event, following the modulation of oxidative stress that,in turn induced a redistribution of both HSP27 and vimentin and caused differentiation of Caco-2 cells into enterocytes, indicating a dose-dependent shift from antioxidant to pro-oxidant effects. However, resveratrol substantially increased iNOS expression. To delineate the mechanisms that underlie the pol-mediated overexpression of p21, we tested the activation of ERK, that is an important regulators of p21 in other cell lines [47]. In growing Caco-2 cell lines polydatin and resveratrol significantly increased ERK1/2 phosphorylation and induced caspase-3 activated apoptosis; these effects, which reached greatest intensity after 24 h treatment, were paralleled by concomitant reduction in cell numbers. Reductions in cell viability elicited by both these compounds were also associated with cell structural changes, possibly caused by cytoskeleton rearrangements, which could have been responsible for loss of cell survival signals. This implies that cell death was probably preceded by relevant cellular alterations affecting cell survival and proliferation. Moreover, we hypothesized that the cause of ISBs-induced cell death was apoptosis, as suggested by activation of caspase-3, acting as a common effector pathway for apoptotic processes originating at both cell membrane and mitochondrial levels [48]. Promotion of cell death by ERK activation may result from the suppression of survival signalling pathways. The phosphatidylinositol 3-kinase/Akt pathway plays a critical role in the regulation of cell survival, and most growth and survival factors activate this pathway [49]. Recently, it was reported that withdrawal of soluble survival factors from primary cultures of mouse renal proximal tubular cells led to ERK1/2 activation that was accompanied by a gradual decrease in Akt activity and apoptosis [50]. It has been proposed that differentiated Caco-2 cell line may be considered an appropriate model for normal colon cells due to its ability to acquire the phenotype of mature small intestinal cells [51,52]. We have found that polydatin has a good profile in terms of cell selectivity, in fact it had a similar and potent cytotoxic activity against the growing Caco-2 cell lines, while it was about 3-fold less potent in differentiated Caco-2 cells. The ability of polydatin to shift the undifferentiated Caco-2 cells to differentiated enterocytes and then undergo a process of programmed cell death, strongly suggests that this compound should be additionally investigated for its potential use in new combination chemotherapy for colon cancer.

## Abbreviations

NO: Nitric oxide; TBA: 2-thiobarbituric acid; CAT: Catalase; ROS: Reactive oxygen species; SOD: Superoxide dismutase; iNOS: Inducible nitric oxide synthase; HE: Hydroethidine.

## Competing interests

The authors declare that they have no competing interests.

## Authors' contributions

SDM, SI, LA, CM, AN, CM, RGP and PS have critically revised the manuscript and have made substantial contributions to conception. All authors have read and approved the final manuscript.
